# Infection and Activation of B Cells by Theiler’s Murine Encephalomyelitis Virus (TMEV) Leads to Autoantibody Production in an Infectious Model of Multiple Sclerosis

**DOI:** 10.3390/cells9081787

**Published:** 2020-07-27

**Authors:** Young-Hee Jin, Charles X. Kim, Jocelin Huang, Byung S. Kim

**Affiliations:** 1 Department of Microbiology-Immunology, Northwestern University Feinberg School of Medicine, Chicago, IL 60611, USA; jhuang10@umphysicians.umn.edu; 2 KM Application Center, Korea Institute of Oriental Medicine, Daegu 41062, Korea; 3 Center for Convergent Research of Emerging Virus Infection, Korea Research Institute of Chemical Technology, Daejeon 34114, Korea; 4 Department of Medicine, Northwestern University Feinberg School of Medicine, Chicago, IL 60611, USA; ckim10@umphysicians.umn.edu; 5 M Health Fairview Heart Clinic, University of Minnesota Health, Edina, MN 55435, USA; 6 M Health Cancer Care, University of Minnesota Health, Edina, MN 55435, USA

**Keywords:** infectious immunity, virus, autoantibodies, demyelination, multiple sclerosis

## Abstract

Theiler’s murine encephalomyelitis virus (TMEV) induces immune-mediated inflammatory demyelinating disease in susceptible mice that is similar to human multiple sclerosis (MS). In light of anti-CD20 therapies for MS, the susceptibility of B cells to TMEV infection is particularly important. In our study, direct viral exposure to macrophages and lymphocytes resulted in viral replication and cellular stimulation in the order of DCs, macrophages, B cells, and T cells. Notably, B cells produced viral proteins and expressed elevated levels of CD69, an activation marker. Similarly, the expression of major histocompatibility complex class II and costimulatory molecules in B cells was upregulated. Moreover, TMEV-infected B cells showed elevated levels of antigen-presenting function and antibody production. TMEV infection appeared to polyclonally activate B cells to produce autoantibodies and further T cell stimulation. Thus, the viral infection might potentially affect the outcome of autoimmune diseases, and/or the development of other chronic infections, including the protection and/or pathogenesis of TMEV-induced demyelinating disease.

## 1. Introduction

Infection with Theiler’s murine encephalomyelitis virus (TMEV) induces a chronic progressive demyelinating disease in susceptible mouse strains, such as SJL/J [1]. It has been well established that the pathogenesis of this virus-induced demyelinating disease is immune-mediated, with the primary involvement of Th1 type CD4^+^ cells [2,3]. Infiltration of proinflammatory T cells is associated with tissue destruction and demyelination [4,5]. However, studies have indicated that the level of Th1 responses during early viral infection is critically important in the protection from, rather than the pathogenesis of, the demyelinating disease [6,7]. Similarly, levels of antiviral antibody responses, as well as antiviral CD8^+^ T cell responses during the early stage of viral infection, play a crucial protective role [8,9,10], whereas their role in later stages of viral infection is less clear. In contrast, studies have indicated that Th17 CD4^+^ T cells are associated with the pathogenesis of TMEV-induced demyelinating disease [11,12]. Since the virus-specific adaptive immune response levels are often closely associated with the initial innate immune responses, it was proposed that the initial innate immunity levels might affect the levels of adaptive immune responses during the early stage of viral infection [13,14,15].

To stimulate and sustain inflammatory T cell responses, several important factors are necessary. First, viral persistence appears to be a critical factor for pathogenesis by providing lasting antigenic stimulation driving high T cell levels [16]. Macrophages, dendritic cells (DCs), and central nervous system (CNS)-resident glial cells from resistant and susceptible mice differentially support the replication of TMEV, and it is suspected that these cell types are involved in viral persistence and immune responses during viral infection, leading to immune-mediated demyelinating disease [13,16]. Professional (DC and macrophages) and non-professional (microglia and astrocytes) antigen-presenting cells (APCs) also play a pivotal role in inducing T cell responses via differential cytokine production [13,15,16]. However, interactions between the virus and B cells during the initial stages of viral infection in the CNS, which leads to inflammatory demyelinating disease, are not clear. Thus, determining whether B cells are permissive to viral infection and defining the role of virus-infected B cells is crucial to understanding not only the pathogenic mechanisms of this virally induced demyelinating disease, but also the potential role of B cells in autoimmune diseases following viral infections in general.

Previous studies have demonstrated the infectivity of TMEV to macrophages and resident glial cells, including microglia, astrocytes, and oligodendrocytes [16,17,18,19]. Additionally, the ability of TMEV to upregulate the gene expression of select chemokines and cytokines in astrocytes and other glial cells has been shown [20,21,22]. The cytokine gene activation following TMEV infection appears to be primarily mediated via toll-like receptor (TLR)3 by viral double-stranded (ds)RNA, replication intermediates of the TMEV genome [23,24]. However, it is conceivable that other TLRs, retinoic acid-inducible gene 1/melanoma differentiation-associated protein (MDA)-5, and protein kinase R may also be secondarily involved in cytokine gene activation, depending on the cell type [25,26]. It is interesting to note that type I interferons (IFNs), along with other cytokines (e.g., interleukin [IL]-12 and IL-6) associated with Th1, Th2, and Th17 differentiation were differentially induced in professional and non-professional APCs of resistant and susceptible mice [11,15]. The cells from resistant mice poorly supported viral infection and cytokine production, promoting protective Th1 responses, whereas cells from susceptible mice vigorously supported viral infection and the production of various proinflammatory cytokines, such as IFN-α/β, IL-1, and IL-6, promoting pathogenic Th17 responses [15,16]. Furthermore, the expression of various costimulatory and major histocompatibility complex (MHC) molecules on the surface of these cells is altered following viral infection, potentially promoting a directional activation of Th cell types, which likely contributes to the differential protective vs. pathogenic functions [11,13]. Therefore, the initial innate responses to viral infection appear to dictate the consequent type and level of adaptive immune responses.

Despite these extensive studies with professional APCs (macrophages and DCs) as well as CNS-resident glial cells, the effect of TMEV infection on immune cells, such as T and B cells, themselves still remains to be determined. In particular, B cells are known as an important professional APC type involved in the activation of Th cell types [27,28], and they also directly produce potent antiviral antibodies. Furthermore, studies indicate that both T and B cells could be modulated or activated by certain TLR-mediated signals [29,30]. B cells are readily activated by several TLR-mediated signals, such as TLR7, TLR8, and TLR9 [31,32,33]. Since TMEV contains a single RNA genome that is a potent ligand for TLR7 [34], as well as producing dsRNA of a replication intermediate, it is conceivable that TMEV infection may lead to B cell activation, similar to DCs and macrophages [13,15,23]. However, an earlier study based on binding and infection using established cell lines suggested that B cells might not be permissive to TMEV infection [35]. Despite the initial study with an established B cell line, it remains unknown whether the primary B cells are permissive to this virus, and whether viral infection activates the B cells to stimulate a particular T cell type and antibody production. The potential involvement of B cells in the pathogenesis of TMEV-induced demyelinating disease is particularly important, because this disease is considered a relevant model for multiple sclerosis (MS) [36], and studies have indicated that treatment of MS patients with antibodies to CD20 B cells is an efficient therapeutic tool [37,38,39].

In this study, we reported that TMEV infects B cells and consequently induces B cell activation. We believe that this is establishing that TMEV infects and activates B cells polyclonally. TMEV infection led to upregulated expression of CD69, an early activation marker, in infected B cells. In addition, the expression of MHC class II and costimulatory molecules was elevated. Moreover, the virus-infected B cells showed elevated antigen-presenting function and antibody production against both self- and non-self-antigens. Interestingly, virus-infected B cells formed a germinal center-like structure in CNS near T cells and promoted the development of pathogenic Th17 cells. Furthermore, autoantibody production was markedly accelerated in autoimmunity-prone mice following TMEV infection. These results strongly suggest that transient viral infection can induce a set of innate immune responses via TLRs, which trigger the activation of B cells and antibody production, including autoantibodies, as well as triggering activation of pathogenic T cells.

## 2. Materials and Methods

### 2.1. Animals

We purchased 4- to 6-week-old female SJL/J, C57BL/6, and BALB/c mice from either the Jackson Laboratory (Bar Harbor, ME, USA) or Charles River Laboratories (Boston, MA, USA) through the National Cancer Institute (Bethesda, MD, USA). Type I IFN receptor knockout (IFN-IR KO) mice were kindly provided by Michel Aguet (University of Zürich, Zürich, Switzerland) via Herbert Virgin (Washington University, St. Louis, MO, USA). Female 129S2/SvPasCrl (129S2/SP) mice were purchased from Charles River Laboratories (Frederick, VA, USA) and used as controls for the IFN-IR KO mice. Female and male NZB×NZW F1 (NZB/WF1) and BXSB/MpJ mice were purchased from The Jackson Laboratory. The mice were housed at the Animal Care Facility of Northwestern University, and experimental procedures approved by the Institutional Animal Care and Use Committee were used.

### 2.2. Splenic Cell Separation

Splenic B and T cells as well as DCs and macrophages were isolated using magnetic beads coated with antibodies to CD19, CD4, CD11c, and CD11b, respectively. MACS^®^ (Miltanyl Biotec, Bergisch Gladbach, Germany) for mouse B cells, T cells, DCs, and macrophages was used as per the manufacturer’s instructions.

### 2.3. Virus Preparation and Viral Infection of Mice

TMEV strain BeAn 8386 was used for intracerebral infection of the two mouse strains, and was propagated in baby hamster kidney (BHK)-21 cells grown in Dulbecco’s modified Eagle medium (DMEM), supplemented with 7.5% donor calf serum. Approximately 30 μL of TMEV (1 × 10^6^ plaque-forming unit [pfu]) was injected into the right hemisphere of 5- to 7-week-old mice anesthetized with isoflurane.

### 2.4. Immunization

Mice were subcutaneously immunized with 25 μg proteolipid protein (PLP)139–151 (HSLGKWLGHPDKF) (Genemed Synthesis, San Francisco, CA, USA) in 100 μL modified complete Freund’s adjuvant (CFA) (Difco, Detroit, MI, USA). Mice were subcutaneously immunized 5 d prior to viral infection with 25 μg of ovalbumin (OVA), which was emulsified 1:1 in CFA (Difco, Detroit, MI, USA).

### 2.5. Plaque Assay

After cardiac perfusion with Hank’s balanced salt solution, the mouse brain and spinal cords were removed. The tissues were homogenized in phosphate-buffered saline (PBS) as a 10% (*w*/*v*) solution using a tissue homogenizer and clarified by low-speed centrifugation (600× *g*). A standard plaque assay was performed using BHK-21 cell monolayers [40]. Plaques in the BHK monolayer were visualized by staining with 0.1% crystal violet solution after methanol fixation.

### 2.6. Infection-Center Assay

A modified infection-center assay from a previously described method [41] was used. Briefly, approximately 3 × 10^5^ BHK cells per well were seeded in 35 mm culture dishes (Corning Costar, Brumath, France) 24 h in advance. A series of 1:10 dilutions of various cell types were incubated with TMEV (multiplicity of infection [MOI] = 10) for 18 h in the presence of DMEM containing 1% bovine serum albumin and 100 U/mL of penicillin–streptomycin. The cultured cells were harvested with trypsin-EDTA and washed three times with PBS. Then, 200 µL of 10-fold dilutions of TMEV-infected cells were seeded in individual wells containing BHK monolayers. The plates were incubated at 37 °C with 5% CO_2_ for 1 h with gentle rocking before overlaying with agarose and further incubating for 5–6 days to develop the viral plaques. The plaques were visualized after methanol fixation, followed by staining with crystal violet.

### 2.7. Antibodies and Flow Cytometry

The following fluorochrome-labeled antibodies were used for flow cytometry: CD4 (clone RM4-5), CD8 (clone 53-6.7), B220 (clone RA3-6B2), CD19 (clone 1D3), CD69 (clone H1.2F3), CD20 (clone 2H7), CD23 (clone B3B4), CD27 (clone LG.3A10), H-2KS (clone KH49), CD62L (clone MEL-14), I-A (clone KH116), CD80 (clone 16-10A1), CD86 (clone GL-1), and CD40 (clone HM40-3). All antibodies were purchased from BD Biosciences (San Diego, CA, USA), unless stated otherwise. The mononuclear cells isolated from the CNS or spleens of TMEV-infected mice were used to analyze the surface expression of various molecules using specific antibodies. The Fc receptors on lymphocytes were first blocked by incubating the cells in 50 µL of 2.4G2 hybridoma supernatant (ATCC, Rockville, MD, USA) for 30 min at 4 °C. The cells were then incubated with the respective antibodies in 50 µL of staining buffer (PBS containing 1% fetal bovine serum and 0.9% sodium azide) for another 30 min at 4 °C, washed twice with the staining buffer, and analyzed on a FACSCalibur flow cytometer (BD Biosciences, San Diego, CA, USA).

### 2.8. Immunofluorescent Staining

SJL mice at 30 d post-infection were perfused with 50 mL of PBS via intracardiac puncture. The brain and spinal cords were dissected and fixed in 4% formalin in PBS for 72 h, transferred into 30% sucrose for an additional 24 h, embedded in paraffin, and sectioned at 6 µm. Then, sections from each animal were deparaffinized, rehydrated, and evaluated by immunofluorescent examination. Briefly, the cross sections of the spinal cord were deparaffinized and subjected to antigen retrieval in pH 6.0 citrate buffer in the microwave. After blocking with 20% normal horse serum in PBS-Triton X-100 for 1 h, the cross sections were stained using rabbit polyclonal anti-TMEV antibody, and Alexafluor 488-labeled goat anti-rabbit immunoglobulin (Ig) G (Invitrogen, Carlsbad, CA, USA) was utilized as the secondary antibody (green). CD20^+^ B cells were stained with rat anti-mouse CD20 antibody, followed with Alexafluor 555-labeled (red) or Alexafluor 488-labeled goat anti-rat IgG (green); CD3^+^ T cells were stained with rat anti-mouse CD3 antibody, followed with Alexafluor 555-labeled (red) goat anti-rat IgG; and the nuclei were counterstained with 4′,6-diamidino-2-phenylindole (DAPI; Sigma, St. Louis, MO, USA), according to the manufacturer’s instructions. The sections were observed and recorded using a Leica DMR fluorescent microscope (Leica Microsystems, Wetzlar, Germany), and images were captured using an AxioCam MRc camera and AxioVision imaging software (Carl Zeiss, Göttingen, Germany).

### 2.9. Quantitative Reverse Transcription-Polymerase Chain Reaction (PCR)

Total cellular RNA was isolated using Trizol^®^ (Invitrogen, Carlsbad, CA, USA). First-strand cDNA was synthesized from 1 µg of total RNA utilizing SuperScript^®^ III First-Strand Synthesis SuperMix (Invitrogen, Carlsbad, CA, USA) at 55 °C. The relative concentrations of cDNA were equalized among the groups based upon the level of GAPDH amplification (35 cycles) by PCR. Message levels were assessed by PCR amplification using the following sense and anti-sense primers: IL-6 (5′-AGTTGCCTTCTTGGGACTGA-3′ and 5′-TCCACGATTTCCCAGAGAAC-3′), IFN-α (5′-ACCTCCTCTGACCCAGGAAG-3′ and 5′-GGCTCTCCAGACTTCTGCTC-3′), IFN-β (5′-GGAAAGATTGACGTGGGAGA-3′ and 5′-CTGAGGCATCAACTGACAGG-3′), and GAPDH (5′-AACTTTGGCATTGTG-GAAGG-3′ and 5′-ACACATTGGGGGTAGGAACA-3′.

### 2.10. Proliferation Assay

CD4^+^ T cells were isolated from indicated mice at 8 d post-infection (dpi). Then, the isolated CD4^+^ T cells (5 × 10^5^/well) were incubated with varying numbers of isolated B cells infected in vitro with TMEV for 24 h in the presence of UV-inactivated TMEV (12.5 µg/mL) for 72 h. Triplicate cultures were pulsed with 1 µCi [^3^H]-thymidine for 18 h, and then [^3^H]-TdR incorporation was determined in a liquid scintillation counter. Data were expressed as mean cpm ± standard error of the mean (SEM).

### 2.11. Enzyme-Linked Immunosorbent Assay (ELISA) for Cytokines and Anti-TMEV Antibodies

Mouse IFN-γ enzyme-linked immunosorbent assay (ELISA) kit was purchased from BD Biosciences, and IL-13 and IL-17 ELISA kits were purchased from R&D Systems, Inc. (Minneapolis, MN, USA). Cytokine levels in the culture supernatants of lymph node and spleen cells were assessed according to the manufacturer’s manual. Briefly, diluted samples were incubated for 2 h with the plate-bound capture antibody. Cytokine expression levels were visualized by horseradish peroxidase (HRP)-conjugated detection antibody in the presence of 3,3′,5,5′-tetramethylbenzidine-1 substrate (BioFX Laboratories, Owings Mills, MD, USA), and absorbance at 450 nm was measured. The anti-TMEV antibody response was determined with ELISA, using UV-TMEV-coated plates, starting with 1/100-diluted serum samples from infected animals as described elsewhere [42].

### 2.12. Western Blot

The uninfected control and virus-infected BHK cells were disrupted using lysis buffer (tris-buffered saline with 1% Triton X-100). The samples (10 µg/lane) were separated by electrophoresis on 12% sodium dodecyl sulfate-polyacrylamide gels, transferred onto a nitrocellulose membrane (Amersham Pharmacia Biotech, Piscataway, NJ, USA), and then treated with anti-myelin basic protein (MBP) antibodies (Santa Cruz Biotechnology, Dallas, TX, USA). HRP-conjugated antibodies were subsequently applied as secondary antibodies. Specific protein levels were visualized using an ECL kit (Amersham Biosciences, Piscataway, NJ, USA).

### 2.13. Autoantibody Detection

The levels of IgG class autoantibodies to single-stranded (ss) DNA, dsDNA, histone, and nucleosome (histone–DNA complex) in mice sera were measured by ELISA, as previously described [43,44]. Briefly, 96-well plates were coated with 50 µg/mL calf thymus dsDNA (Sigma) (sheared by sonication and digested with S1 nuclease [Promega, Madison, WI]), 50 µg/mL ssDNA (prepared by boiling dsDNA and rapid chilling), and 10 µg/mL histone (Sigma) or nucleosome (histone and dsDNA) in PBS overnight at 4 °C. Then, the 1/100 diluted sera in PBS-10% horse serum were added and incubated overnight at 4 °C. Finally, anti-IgG antibody conjugated to alkaline phosphatase (Sigma) diluted to 1/3000 was added, and the plate was incubated at room temperature for 1 h. The p-nitrophenylphosphate substrate (Sigma) was added, and the absorbance at 405 nm was read with an ELISA plate reader (Molecular Devices, Sunnyvale, CA, USA).

### 2.14. Statistical Analysis

Data are either shown as the mean ± standard deviation (SD) of 2–3 independent experiments, or as one representative example from at least three independent experiments. Multiple group comparisons were performed by one-way analysis of variance (ANOVA) with the Tukey–Kramer post hoc analysis. The significance of differences in the mean values was determined by Student’s t-test (two-tailed) using GraphPad Prism software version 6.0 (GraphPad Software, Inc., San Diego, CA, USA) unless otherwise indicated. *p* < 0.05 was considered statistically significant.

## 3. Results

### 3.1. Viral Infection and Replication Levels in Different Cell Types of Splenic Cell Cultures

**Figure 1:**
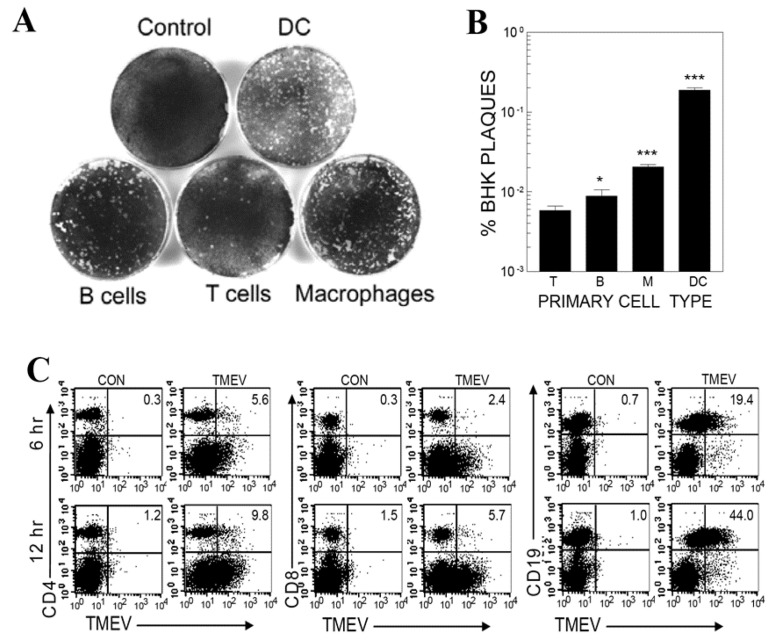
Levels of viral replication in different primary splenic cell types. (**A**) Isolated primary splenic dendritic cells (DC), B cells, T cells, and macrophages (M) were infected with Theiler’s murine encephalomyelitis virus (TMEV) (MOI = 10) for 24 h, and then virus levels were assessed by infectious center assay on baby hamster kidney (BHK) monolayers. (**B**) The results of the infectious center assays of these cell types were compared with those of the levels of plaques in BHK cells infected with TMEV for 24 h. * *p* < 0.05; *** *p* < 0.001. (**C**) The levels of viral protein production in CD4^+^ and CD8^+^ T cells and B cells were determined by using green fluorescent protein (GFP) expression after infection with GFP-TMEV for 6 and 12 h. The GFP expression was assessed by flow cytometry in conjunction with CD4, CD8, and CD19 expression. The presented data are representative of three independent experiments.

It was previously shown that primary glial cells (neurons, astrocytes, and microglia) are permissive to TMEV infection and support viral replication, leading to viral persistence in the CNS [17,21,45,46]. Furthermore, professional APCs, such as DCs and macrophages, are also permissive to TMEV infection [13,15]. However, it has not been clear whether any B cell types are permissive to viral infection and support viral replication. This information may be critically important in understanding the potential pathogenic mechanisms involved in TMEV-induced demyelinating disease, because this cell type is unique for both antibody production and T cell stimulation. To compare the relative permissiveness of B cells to TMEV infection, primary B cells and other cell types (T cells, macrophages, and DCs) were isolated from the spleens of naïve SJL mice and then infected in vitro with TMEV (MOI = 10). Subsequently, the viral replication levels in these cell types after 24 h were assessed by a sensitive infection-center assay on BHK monolayers. Varying degrees of viral replication in these cell types were observed in the order of primary DCs, macrophages, B cells, and T cells (Figure 1A,B). The viral replication levels in the primary T cells were extremely low and negligible, but the levels in macrophages and DCs were relatively high, although the levels were detected in <20% of BHK cells. The results with macrophages and DCs were consistent with the previous reports [13,15]. However, it was surprising to note that B cells showed a substantial level of viral replication. Further analysis of bulk spleen cells infected with green fluorescent protein (GFP)-TMEV for 6 or 12 h confirmed the resistance of CD8^+^ and CD4^+^ (<5% and <9%, respectively, at 12 h) T cells (Figure 1C). In contrast, as much as 43% of B cells produced GFP and viral proteins, indicating TMEV replication in the B cells (Figure 1C). The GFP RNA sequence was inserted into TMEV of GFP-TMEV, so the production of GFP protein indicated a productive viral infection [47]. Therefore, these results clearly indicated that B cells were a major cell population in the peripheral lymphoid organs, which were permissive to TMEV infection and supported viral replication. These results strongly suggest that B cells may also play an important role in the protection and/or pathogenesis of TMEV-induced demyelinating disease, since B cells are considered important specialized APCs producing critical effector molecules, anti-viral antibodies.

### 3.2. Subpopulation of B Cells Permissive to TMEV Infection

**Figure 2:**
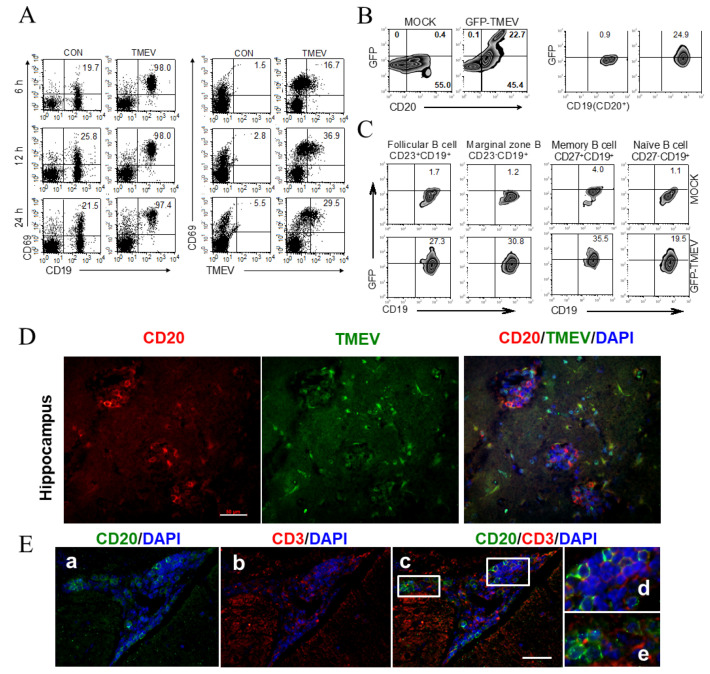
Characterization of B cells productively infected with TMEV. (**A**) Spleen cells were infected with GFP-TMEV for 6, 12, and 24 h. B cells were then analyzed for their activation by the expression of CD69 and CD19 as well as CD69 and GFP of CD19^+^ gated cells using flow cytometry. (**B**) Spleen cells were infected with GFP-TMEV for 24 h and then analyzed for their expression of GFP molecules in conjunction with CD20^+^ and CD19^+^ B cells. (**C**) B cell subpopulations permissive to TMEV infection were examined using antibodies to CD23, CD27, and CD19. CD23^+^CD19^+^ cells were considered as follicular B cells, CD23^−^CD19^+^ cells as marginal zone B cells, CD27^+^CD19^+^ cells as memory B cells, and CD27^−^CD19^+^ cells as naïve B cells. Immunofluorescent staining of the brain of SJL mice infected with TMEV (D and E). (**D**) Brain sections of TMEV-infected mice at 14 dpi were stained with rabbit anti-TMEV antibody (green) and rat anti-mouse CD20 antibody (red) and counterstained with 4′,6-diamidino-2-phenylindole (DAPI) (blue). There were clustered CD20^+^ B cells in the brain. Scale bar = 50 µm (**E**) Immunofluorescent staining of CD20^+^ cells (green) and CD3^+^ T cells (red) and counterstaining with DAPI (blue) in the cerebellum of SJL mice infected with TMEV at 30 dpi. Insets of (c) are shown in (d) and (e) in a larger scale. Scale bar = 50 µm. The presented data are representative of three independent experiments (*n* = 3).

The activation of B cells, like T cells, results in the upregulation of CD69, an early activation marker, preceding antibody production [48,49]. To examine the consequences of viral infection in primary B cells, the expression of CD69 and viral proteins in CD19^+^ cells were analyzed at 6 h, 12 h, and 24 h post-infection using flow cytometry (Figure 2A). A drastically increased level of CD69 (>97% of CD19^+^ cells) was observed as early as 6 h after TMEV infection compared with 20–26% in uninfected cells. The peak level (up to 37% of CD69^+^ cells) of viral proteins was detectable at 12 h post-infection, suggesting that the upregulation of CD69 on B cells after TMEV infection preceded viral protein production.

CD20 is known as B-lymphocyte surface antigen and is expressed on almost all B cells, except plasma cells. The level of CD20 expression increases with B cell maturation, differing in expression levels in the B cell subsets [50]. Approximately 23% of CD20^+^ cells and 25% of CD19^+^CD20^+^ cells displayed GFP after GFP-TMEV infection, indicating that these cells were productively infected with TMEV (Figure 2B). Further flow cytometry experiments showed that TMEV infected similar levels (27% vs. 31%, respectively) of CD23^+^CD19^+^ cells, representing follicular B cells, and CD23^−^CD19^+^ cells, representing marginal zone B cells (Figure 2C). However, TMEV infected nearly two-fold higher levels of CD27^+^CD19^+^ memory B cells, compared with CD27^−^CD19^+^ naïve B cells. Therefore, it appeared that memory B cells were more permissive to TMEV than naïve B cells, correlating with previous findings that activated macrophages were more permissive to viral infection than naïve macrophages [47,51].

The infection level of B cells in the CNS of TMEV-infected mice was further assessed using immunofluorescent staining of the brains of SJL mice infected with TMEV. At 14 dpi, co-staining with rabbit anti-TMEV antibody and anti-mouse CD20 antibody was observed, suggesting that many clustered, activated, and mature CD20^+^ B cells produced viral antigens indicative of TMEV infection (Figure 2D). These results indicated that mature B cells were also productively infected with TMEV and produced viral proteins in the infected B cells in the CNS of TMEV-infected mice. Further staining in conjunction with the anti-CD3 antibody strongly suggested that T cell populations were also adjacent to the TMEV-infected B cell clusters (Figure 2E). These results suggested that TMEV-infected B cells formed a germinal center-like structure containing T cell components in the CNS of TMEV-infected SJL mice.

### 3.3. Cytokine Gene Activation in B Cells upon TMEV Infection

**Figure 3:**
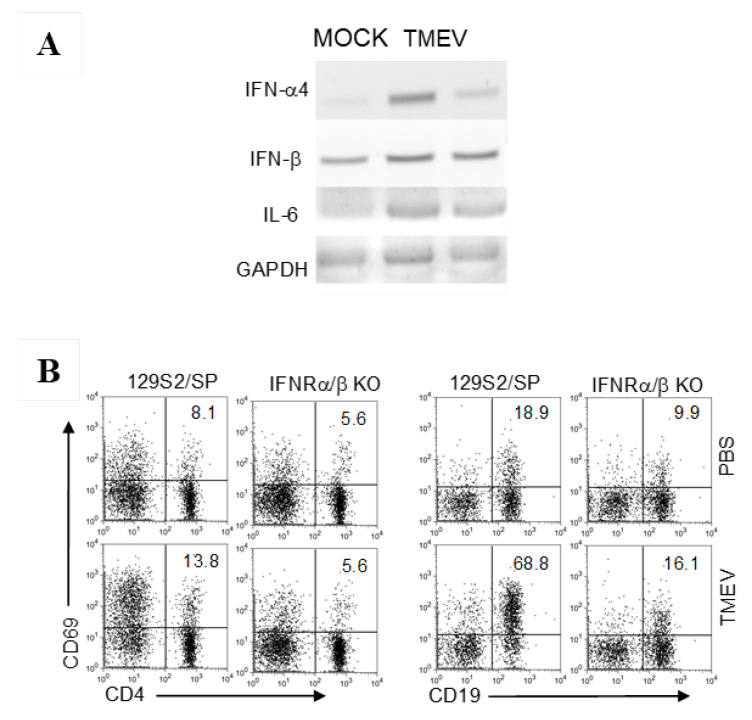
Select cytokine gene activation in B cells upon TMEV infection. (**A**) Isolated splenic B cells were infected with two different stocks of TMEV (MOI = 2) for 48 h, and then the levels of IFN-α4, IFN-β, and IL-6 were determined using RT-PCR. (**B**) The levels of an activation marker (CD69) of CD4 and CD19 in TMEV-infected or uninfected IFNα/βR-deficient and control 129S2 mice were determined using flow cytometry. The presented data are representative of three independent experiments (*n* = 5).

To further investigate the extent of B cell activation, we assessed the expression levels of various cytokine genes that were activated following TMEV infection. Further assessments using conventional PCR assays indicated that the expression of type I IFNs, and IL-6 were also upregulated in the B cells (Figure 3A). These results clearly indicated that TMEV infection activated B cells to produce various cytokines, similar to B cells activated by lipopolysaccharide or specific antigens [52,53]. It is known that type I IFNs are produced upon TMEV infection via various pattern recognition receptors and these cytokines activate different APCs [54,55,56], so we further examined the role of type I IFNs on B cell activation following TMEV infection using IFNα/β receptor (R)-sufficient and deficient mice (Figure 3B). CD19^+^ B cells from IFNα/βR-sufficient mice displayed a significant activation (~70% CD69^+^) upon TMEV infection compared with the CD19^+^ B cells (16% CD69^+^) from IFNα/βR-deficient mice, suggesting the involvement of IFNα/β in B cell activation. This profoundly different activation of B cells was in contrast to the T cells (14% vs. 6%, respectively), which showed minimal activation.

### 3.4. Upregulated Expression of Costimulatory Molecules on B Cells after TMEV Infection

**Figure 4:**
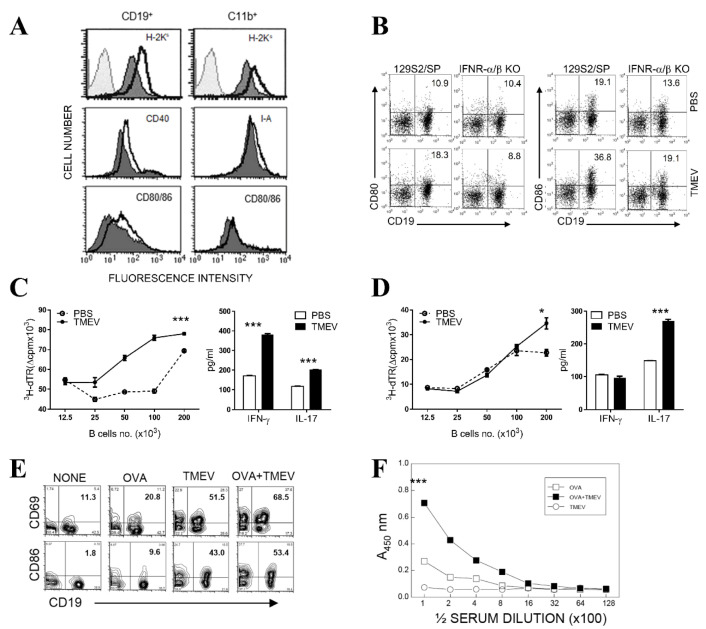
Upregulation of cytokine production and costimulatory molecule expression following TMEV infection. (**A**) The levels of major histocompatibility complex (MHC) and costimulatory molecules on CD19^+^ B cells, and CD11b^+^ macrophages were determined by flow cytometry following infection with TMEV for 24 h. Gray line-colored histogram, isotype control; black line open histogram, TMEV-infected cells; and black line-colored histogram, mock-infected cells. (**B**) Costimulatory molecules on B cells of spleen from normal and IFN-α/βR knockout mice were determined by flow cytometry following infection with TMEV for 24 h. (**C**) SJL splenic CD4^+^ T cells from PLP_139–151_-primed SJL mice at 10 d post-immunization were stimulated with B cells from naïve SJL mice after infection with TMEV or mock-infection (PBS) for 24 h in the presence of the immunizing PLP_139–151_ peptide (2 μM) (left panel). The proliferation rate was determined by [^3^H]-thymidine uptake assay, and the values in the absence of PLP_139–151_ were subtracted (right panel). Cytokine levels (interferon (IFN)-γ and IL-17) in the culture supernatants of PLP_139–151_-primed T cells in the presence of 1 × 10^5^ mock-infected or TMEV-infected B cells were assessed using specific enzyme-linked immunosorbent assay (ELISA). (**D**) BALB/c splenic CD4^+^ T cells were stimulated with B cells from naïve SJL mice after infection with TMEV or mock-infection (PBS) for 24 h for alloantigen response assay. The proliferation rate was determined by [^3^H]-thymidine uptake assay (left panel), and cytokine levels in the culture supernatants were determined by specific ELISA (right panel). Statistical significance was analyzed by Student’s t-test. * *p* < 0.05; *** *p* < 0.001 (**E**) Costimulatory molecules on B cells of spleen from ovalbumin (OVA)-immunized and/or TMEV-infected SJL mice were determined by flow cytometry. (**F**) Antibody levels against OVA in serum from OVA-immunized and/or TMEV-infected SJL mice were determined by ELISA. The statistical significance between ova and ova+ tmev was analyzed by one-way ANOVA. *** *p* < 0.001. The presented data are representative of three independent experiments.

B cells are also professional APCs to T cells, so we further assessed the expression levels of costimulatory molecules on virus-infected B cells (Figure 4A). The expression levels of MHC antigens and or CD80/86 were compared on primary splenic B cells, and macrophages following TMEV infection. The expression levels of MHC antigens as well as costimulatory molecules (CD80/86 and CD40) were significantly upregulated on B cells and macrophages. These results strongly suggested that B cells, similar to macrophages, were stimulated following TMEV infection. Further examinations of CD80 and CD86 on CD19^+^ B cells from IFN-α/βR-deficient mice indicated that the levels of B cells expressing these costimulatory molecules were ~2× lower in the absence of type I IFN signaling (Figure 4B). Therefore, the upregulation of these costimulation molecules was partially dependent on the presence of type I IFNs induced after TMEV infection, as previously shown with TMEV-infected macrophages [57].

### 3.5. Elevated T Cell-Activating Function of B Cells Following TMEV Infection In Vitro

To further test the effects of the B cell activation following TMEV infection, the antigen-presenting function of TMEV-infected B cells in the T cell stimulation was assessed in vitro (Figure 4C,D). Isolated B cells infected with TMEV in vitro induced more vigorous T cell proliferative responses of CD4^+^ T cells from PLP_139–151_-immunized SJL/J mice against the cognate antigen (PLP_139–151_ peptide), compared with mock-infected B cells (Figure 4C, left panel). Therefore, the upregulated expression of costimulatory molecules on virus-infected B cells appeared to facilitate a more efficient T cell proliferative response to a primed antigen. To further assess the potential alterations in the cytokine profiles of T cells stimulated by TMEV-infected B cells, the levels of T cell cytokines, IFN-γ and IL-17, were measured after stimulation, with control and virus-infected B cells with and without the cognate antigen (Figure 4C, right panel). The production of all these cytokines by PLP_139–151_-primed CD4^+^ T cells was elevated in response to the cognate PLP_139–151_ presented by the virus-infected B cells. These results strongly suggested that virus-infected B cells provided higher T cell stimulation, leading to the elevated production of T cell effector cytokines of Th1 and Th17 cell types.

To further assess the potentially elevated non-specific responses of T cells stimulated by TMEV-infected B cells, levels of proliferation and the key T cell cytokines of allogenic CD4^+^ T cells from BALB/c mice were also measured, following stimulation with mock and TMEV-infected SJL B cells (Figure 4D). The proliferative responses (Figure 4D, left panel) and the production of cytokine IL-17, but not IFN-γ, (Figure 4D, right panel) were elevated in response to CD4^+^ T cells following allogenic stimulation with TMEV-infected B cells. This result was in contrast to the stimulation of antigen-primed syngenic T cells, which showed elevated production of IFN-γ and IL-17 (Figure 4C). The differences between these T cell cytokine patterns might reflect the differences in the Th subtypes from naïve CD4^+^ T cells in allogenic responses vs. peptide-primed CD4^+^ T cells in PLP_139–151_-reactive T cells. It was most likely that virus-infected B cells skewed the differentiation of Th cell subtypes from naïve T cells, compared with the activation of primed CD4^+^ T cells containing differentiated Th subpopulations, as shown previously with TMEV-infected DCs [11].

### 3.6. Elevated Antibody Production in Mice Infected with TMEV

We further examined whether the B cell activation and the ability to enhance T cell stimulation in vitro could also lead to increases in antibody production in vivo (Figure 4E,F). SJL mice primed with OVA in complete adjuvant for 5 d were infected with TMEV, and, at 5 dpi, the levels of B cell activation, costimulatory molecule expression, and antibody production to OVA were compared with those of similarly primed uninfected control mice (Figure 4E). B cells from OVA-immunized mice were activated on the basis of the expression of CD69 and CD86. The B cells from TMEV-infected mice presented CD69 and CD86 at far higher proportions (2× and 4×, respectively), compared with the B cells from uninfected OVA-immunized mice. Interestingly, the highest proportions of B cells from OVA-immunized and TMEV-infected mice displayed these markers, suggesting that antigen priming and TMEV infection independently and synergistically activated B cells.

To further test whether the activated B cells exposed to TMEV infection led to higher levels of antibody production, the levels of OVA-specific antibodies in the sera from OVA-immunized, TMEV-infected, or OVA-immunized and TMEV-infected mice were assessed using ELISA (Figure 4F). The production of antibodies to OVA in mice immunized with OVA together with TMEV infection was elevated several-fold compared with that in mice immunized with OVA without viral infection. Interestingly, mice infected with TMEV alone and without OVA immunization did not produce measurable antibodies against OVA, suggesting that viral infection synergistically stimulated specific antigen-primed B cells, and the presence of specific antigen or antigen priming was necessary for the synergistic stimulation of antibody production.

### 3.7. Reactivity of Serum Antibodies from TMEV-Infected Mice to Normal CNS Components

**Figure 5:**
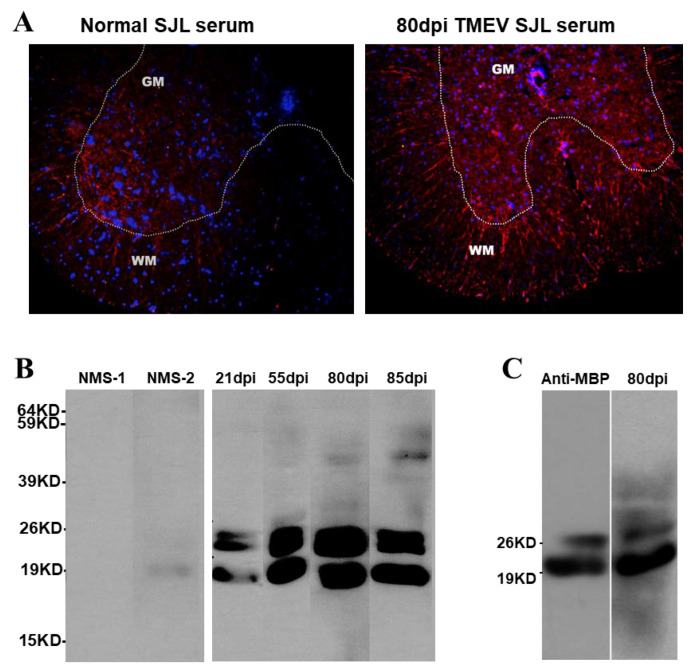
Reactivity of antibodies from TMEV-infected mice to normal spinal cord components. (**A**) Cross sections of normal SJL spinal cords were stained with sera from mice infected with TMEV and control medium. The tissue sections were stained with the mouse sera followed with goat anti-mouse IgG secondary antibody (red), and cell nuclei were counterstained with DAPI (blue). There were some positive cells in the sections stained with 80 dpi serum compared with the cells stained with normal mice serum (NMS). Gray matter (GM), white matter (WM). Scale bar = 50 µm. (**B**) Spinal cord extracts from normal SJL mice were subjected to western blotting to further determine the reactivity of sera from normal uninfected and TMEV-infected SJL mice at various time points. (**C**) The immunoreactivity pattern of the spinal cord extract with sera from TMEV-infected mice was compared with the reactivity to commercial anti-MBP antibodies. The presented data are representative of at least three separate experiments.

We further examined the possibility that mice chronically infected with TMEV produced antibodies to autoantigens in the CNS, which were released following tissue damages as the consequences of viral infection and B cell activation. Immunofluorescence staining of normal spinal cord sections treated with sera from normal mice or TMEV-infected mice at 80 dpi, followed by treatment with PE-labeled goat anti-mouse IgG antibodies (Figure 5A), showed that sera from chronically infected mice with TMEV were clearly reactive to antigens in both the gray and white matter of the normal CNS. However, no reactivity was apparent with the sera from normal mice that were not infected with the virus.

In an attempt to identify some of the CNS components reactive to the sera from TMEV-infected mice, we examined the reactivity of sera from normal and TMEV-infected mice at varying stages of infection with lysates of normal spinal cords using western blotting (Figure 5B). Strong and several weak reactions to three different distinct bands were observed in the sera from TMEV-infected mice as early as 21 dpi. Interestingly, one of the two normal sera tested also showed weak but trace reactivity to one or two bands, suggesting that normal SJL mice produced a low level of the same autoantibodies. It was previously claimed that TMEV infection sometimes results in the production of antibodies reactive to autoantigen, including myelin basic protein (MBP) [58,59], so we further tested whether some of the reactive bands represented MBP by comparing the reactivity to a commercial anti-MBP antibody (Figure 5C). When compared with the bands reactive to the anti-MBP antibody, the major bands reacted with the sera from TMEV-infected mice, representing MBP isoforms. These results were consistent with the previous report that some TMEV-infected mice produce antibodies to MBP [58]. Therefore, the results suggest that chronic TMEV infection led to the production of antibodies to MBP and other autoantigens in the CNS, which might represent autoantigens released following viral infection causing demyelination.

### 3.8. Acceleration of Autoantibody Production in Autoimmune-Prone Mice after TMEV Infection

**Figure 6:**
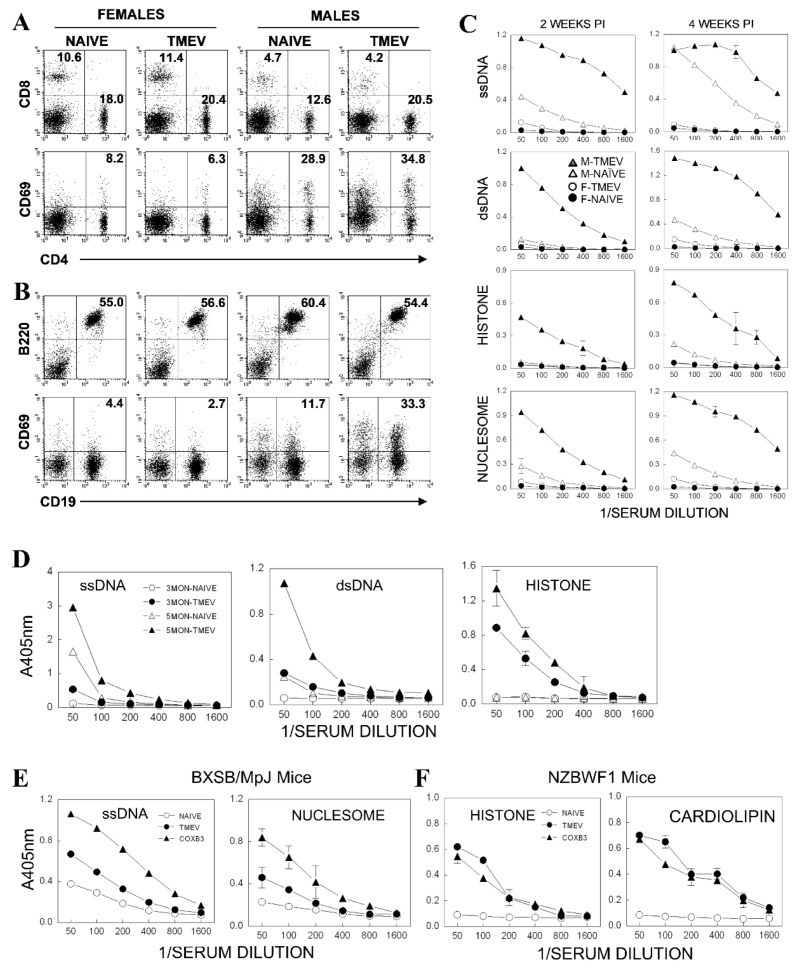
Accelerated production of autoantibodies in systemic lupus erythematosus (SLE)-prone mice infected with TMEV. (A and B) Two-month-old female and male BXSB/MpJ mice were infected intracerebrally with TMEV (5 × 10^6^ pfu). Splenic CD4^+^ T cells (**A**) and CD19^+^ B cells (**B**) were analyzed for the expression of an activation marker (CD69) using flow cytometry at 4 weeks post-infection. (**C**) The levels of indicated autoantibodies (ssDNA, dsDNA, histone, and nucleosome) in pooled sera (*n* = 5) from three mice per group were assessed using ELISA at 2 and 4 weeks post-infection. The statistical significance between M-TMEV and M-NAIVE was analyzed by one-way analysis of variance (ANOVA). *p* < 0.001 (**D**) Ten-week-old NZB/WF1 mice were infected intracerebrally with TMEV (5 × 10^6^ pfu). At 2 weeks post-infection of 3-month-old and 10 weeks post-infection of 5-month-old mice, the levels of autoantibodies to ssDNA, dsDNA, and histone in pooled sera (*n* = 5) from the above groups (three mice per group) were assessed using ELISA. Similarly accelerated production of autoantibodies in the SLE-prone mice infected with closely related CoxB3 virus. The statistical significance between 5MON+TMEV and 5MON+NAIVE was analyzed by one-way ANOVA. *p* < 0.001 (**E**) two-month-old BXSB/MpJ mice were infected with either TMEV or CoxB3, and the autoantibody (ssDNA and nucleosome) levels in pooled sera (*n* = 5) were assessed at 2 weeks post-infection. (**F**) Ten-week-old NZB/WF1 mice were infected with TMEV or CoxB3 virus, and the autoantibodies (histone and cardiolipin) in pooled sera (*n* = 5) were assessed at 2 weeks post-infection. The statistical significance between TMEV/COXB3 and naive was analyzed by one-way ANOVA. *p* < 0.001. The presented data are representative of three independent experiments.

We further examined the possibility of increase in the production of other autoantibodies after TMEV infection using systemic lupus erythematosus (SLE)-prone mice. Male BXSB/MpJ mice, unlike their female counterparts, possess two copies of TLR7 genes and are prone to the spontaneous development of autoantibodies [60,61]. To test whether TMEV infection affected the development of autoantibodies, 2-month-old female and male BXSB mice were infected intracerebrally with TMEV (5 × 10^6^ pfu), and the levels of Th and B cell activation, which are involved in the development of autoimmunity, were compared (Figure 6A,B). The levels of activated CD69^+^ Th cells were 3× higher in male BXSB mice without viral infection and further increased after TMEV infection compared with their counterpart female mice, which showed no difference following viral infection (Figure 6A). Similarly, higher proportions (>2×) of B cells from male mice were activated (CD69^+^) than those from female mice and further increased (~3×) in B cells of male mice after TMEV infection but not in B cells of female mice (Figure 6B). The levels of autoantibodies against ssDNA, dsDNA, histone, and nucleosome were further compared in these groups at 2 and 4 weeks after TMEV infection (Figure 6C). As expected, uninfected male BXSB mice spontaneously developed autoantibodies to these autoantigens compared with female mice displaying undetectable or minimal levels. The levels of the autoantibodies in male mice were increased over time. Interestingly, TMEV-infected male mice showed accelerated development of autoantibodies to these autoantigens, whereas the antibody levels remained very low in infected female mice. These results clearly demonstrated that TMEV infection in autoimmune-prone BXSB male mice markedly accelerated the production of autoantibodies. Male BXSB mice possess additional TLR7 and the TMEV RNA genome is the potential ligand for TLR7, so the viral infection might promote autoantibody production via TLR7-mediated triggering. It was also interesting to note that female mice infected with TMEV eventually showed low but detectable levels of autoantibodies, suggesting that TMEV infection might also trigger autoimmune responses at a much slower rate under conditions of normal TLR7 expression.

Moreover, NZB/WF1 mice develop SLE-like disease spontaneously, and TLR-mediated signaling may affect disease development [62,63]. To examine the effects of TMEV infection on the development of autoantibody responses, antibody levels to ssDNA, dsDNA, and histone in NZB/WF1 mice infected with TMEV at 10 weeks of age were measured when they were 3 and 5 months old (Figure 6D). Uninfected NZB/WF1 3-month-old mice did not show a measurable level of autoantibodies, but at 5 months old, they produced measurable levels of antibodies to ssDNA and dsDNA. In contrast, TMEV-infected NZB/WF1 displayed all the autoantibodies in an accelerated fashion; at 3 months old, the infected mice showed similar levels of antibodies to ssDNA and dsDNA as those of 5-month-old uninfected mice, and a far exceeding level of antibodies to histone. Therefore, the production of autoantibodies to histone was particularly accelerated after TMEV infection in NZB/WF1 mice.

To further examine whether TMEV was unique in the acceleration of autoantibody production in these mice, the levels of autoantibodies in BXSB (Figure 6E) and NZB/WF1 mice (Figure 6F) following infection with TMEV or Coxsackie virus B3 (CoxB3), which belongs to Picornaviridae and induces myocarditis, were examined. The results indicated that both viral infections accelerated the production of autoantibodies in these autoimmune-prone mice compared with the uninfected mice. Interestingly, CoxB3 virus-infected BXSB male mice showed a more elevated autoantibody production than the mice infected with TMEV, although similar accelerations were achieved by these viruses in NZB/WF1 mice. Therefore, the differences observed in the acceleration of autoantibody production might reflect differences in the B cell triggering mechanisms of the different virus types.

## 4. Discussion

It has been previously documented that T cell as well as antibody responses to self-antigens in the CNS were induced during chronic infection of susceptible mice with TMEV [58,59,64]. Consequently, it is unclear whether viral infection-induced autoimmune-like demyelinating disease after chronic TMEV infection represents an infection-induced autoimmune disease or pathogenic immune responses to the viral antigens. Numerous studies have indicated that viral infection, including TMEV, induces innate immune responses via pattern recognition receptors, which affect the consequent adaptive immune responses [23,25,26,65,66]. The innate immune cytokines, including type I IFNs, IL-6, and IL-1, promote inflammatory Th17 responses over protective Th1 responses [11,13,15,16,67]. Despite the many studies focused on the type of Th responses, studies on the effects of these innate immune responses on B cell activation and/or antibody production are scarce because B cells have been considered non-permissive to TMEV infection [35].

In this study, we have demonstrated that primary B cells are permissive to TMEV infection, similar to all other APCs (Figure 1). TMEV infected B cells are activated, leading to the upregulation of costimulatory molecules (Figure 2). Further studies showed that a wide range of B cells was permissive to TMEV infection, including memory B cells and naïve B cells, regardless of the location (Figure 2). In TMEV-infected susceptible mice, germinal center-like structures with infected B cells and activated T cells were observed, particularly in the hippocampus lesion (Figure 2). Furthermore, B cells also produced IFN-α, IL-6, and IL-1 upon TMEV infection (Figure 3). Interestingly, IFN-α/β appeared essential for B cell activation following TMEV infection, as the B cells lacking IFN-α/βR failed to become activated. This was in contrast to the fact that IFN-α/β produced by TMEV infection was shown to induce DC apoptosis and inhibit DC activation, resulting in poor protective T cell responses [13,57]. Nevertheless, these results strongly suggest that virus-infected B cells and other APCs intimately affect the development of T cell types, which are either pathogenic or protective against the development of chronic demyelinating disease. The skewed development of Th type responses in the presence of excessive IL-6 and IL-1 produced upon viral infection have been previously demonstrated with TMEV-infected DCs [11,67]. Similarly, the presence of B cells excessively producing these cytokines might also affect the development of Th cell type responses favoring the pathogenesis of virally induced demyelinating diseases (Figure 4). Additionally, IFN-α/β, IL-6, and IL-1 are known to activate B cells or B cell development [68,69,70].

Our study results indicated that B cells infected with TMEV displayed upregulated expression of costimulatory molecules, such as CD69 and CD80/86, in an IFN-α/β signaling-dependent manner (Figure 3B and Figure 4B). Interestingly, TMEV-infected B cells synergistically stimulated antigen-specific CD4^+^ T cells with strong cytokine production and led to elevated proliferation. In addition, it appeared that viral infection facilitated polyclonal B cell activation and enhanced cognate antibody production by B cells against OVA, which was unrelated to viral antigens (Figure 4E,F). Furthermore, TMEV-infected SJL mice possessed elevated levels of autoantibodies to MBP (Figure 5), of which production was enhanced following viral infection. It was interesting to note that TMEV infection also led to elevated Th cell responses to other CNS autoantigens, such as PLP_139–151_, known as epitope-spreading [64]. Therefore, TMEV infection may stimulate not only B cells but also CD4^+^ T cells reactive to autoantigens, which are generally not activated, because of the antigen sequestration in the CNS. However, neurotropic viral infection may release these autoantigens due to neuroinflammation and apoptosis of infected glia [13,71]. Although the role of immune responses to such CNS autoantigens in the pathogenesis of demyelinating disease remains unelucidated, these immune responses may significantly contribute to the pathogenesis of TMEV-induced demyelinating disease.

To further understand the potential effects of viral infection in the development of other autoimmune diseases, we infected SLE-prone BXSB and NZB/WF1 mice with TMEV and the Coxsackie virus (Figure 6). Both viral infections accelerated the production of autoantibodies in these autoimmune-prone mice. Therefore, TMEV infection did not limit the enhancement of autoimmune responses to the CNS antigens, and appeared universal for the elevation of immune responses to self-antigens (Figure 5 and Figure 6), as well as non-self-antigens (Figure 4). It was previously shown that TLRs contributed to the development of autoimmune diseases in SLE-prone BXSB male and NZB/WF1 mice [60,61,62,63]. Interestingly, infections of these mice with TMEV and the related Coxsackie virus, which are known to activate several TLR-mediated signals, including TLR2, TLR3, and TLR7 [15,23,25,72,73], significantly accelerated the development of autoantibody production. This was consistent with clinical results indicating that microbial infections exacerbate SLE [74,75] and MS [76,77]. Although B cell activation in these SLE-prone mice appeared to be associated with TLR7, other TLRs may also participate in the B cell activation via NF-κB and critical cytokines affecting B cell activation, such as IFN-α/β, IL-1, and IL-6 [78,79,80]. In fact, splenic B cells from SJL mice showed upregulated expression of CD80/86 costimulatory molecules, as well as the CD69 activation marker upon treatment with ligands of TLR2, TLR3, TLR4, TLR7, and TLR9, strongly suggesting that any of these TLRs might be able to stimulate B cells (Figure S1).

**Figure 7:**
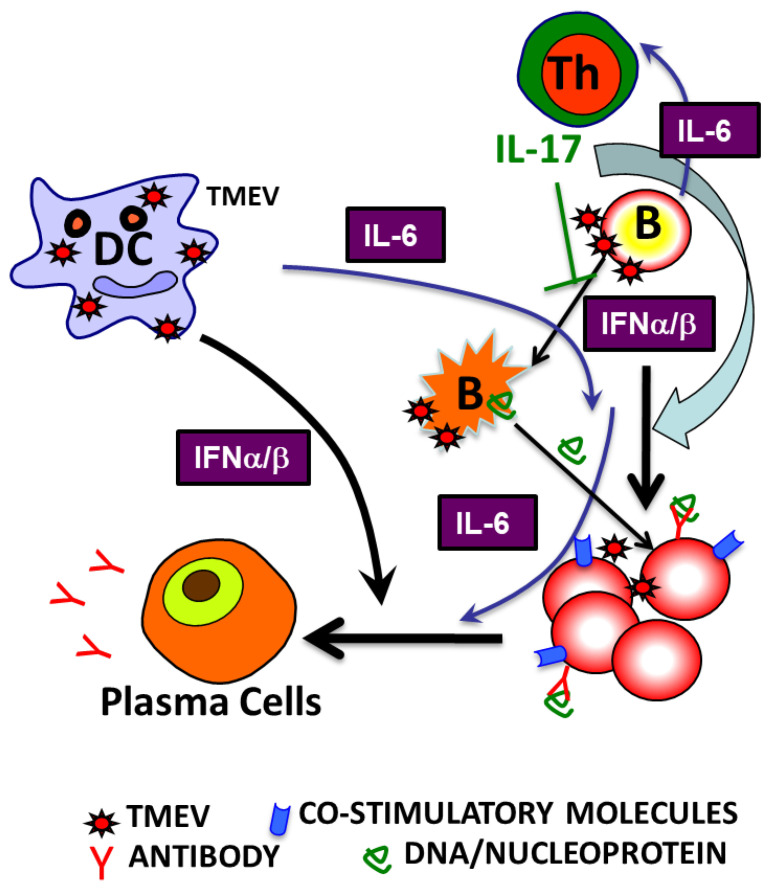
Potential mechanisms underlying polyclonal B cell activation following viral infection. Infection of susceptible mice with neurotropic TMEV induces excessive levels of innate immune cytokines, including type I IFNs, IL-6, and IL-1, which promote inflammatory Th17 responses over protective Th1 responses, leading to high viral loads in the CNS. Various glias and antigen-presenting cells (APCs), including B cells, are permissive to the viral infection and participate in the innate immune responses and viral persistence. Interestingly, B cells are activated and stimulated to produce elevated levels of antibodies. Such high viral loads and innate cytokines as well as adaptive immune responses in the CNS led to CNS tissue damage releasing sequestered autoantigens. Although the role of autoimmune components in the pathogenesis of virus-induced demyelinating disease is unclear, many considerable circumstantial evidences suggest a potentially important role of microbial infection in the induction and/or progression of various autoimmune diseases.

Our results strongly suggest that TMEV infection of APCs, including DCs, macrophages, B cells, and glial cells, induces various innate immune responses via TLRs and other pattern recognition receptors, such as MDA5 and protein kinase R [15,23,25,26]. These innate immune responses trigger the excessive production of IFN-α/β, IL-6, IL-1, and prostaglandin E2, and the overproduction of these cytokines in cells from susceptible mice promotes viral loads and persistence, by dampening protective Th1 cell responses while enhancing pathogenic Th17 responses [11,81]. Furthermore, these innate immune cytokines produced in different glias, DCs, and macrophages may also activate B cells to produce antibodies and trigger T cell responses against sequestered autoantigens, which are released following virus-induced tissue damages (Figure 7). TMEV-infected B cells formed a germinal center-like structure with close proximity to T cells in the CNS (Figure 2), which also helped to stimulate pathogenic T cells to recognize not only viral determinants, but also autoantigens. Antibodies to viral determinants appear to play a minor role in the protection of mice from the pathogenesis of demyelinating disease compared with CD4^+^ T cells and CD8^+^ T cells [8,82]. Nevertheless, the treatment of mice with monoclonal anti-CD20 antibody appears to accelerate the pathogenesis of TMEV-induced demyelinating disease, suggesting a protective role of the B cells [83]. It remains unclear whether these autoimmune responses induced after viral infection play any significant role in the pathogenesis of TMEV-induced demyelinating disease and other viral infections. Additionally, it is unknown whether the protection by the antibodies against viral determinants outweighs the pathogenesis by the CNS autoantibodies. Nevertheless, it is clear that many autoimmune diseases deteriorate after viral infections, including SLE [84,85,86]. Additionally, several autoimmune diseases, including MS, showed that microbial infections often resulted in disease exacerbation [76,77,87], strongly suggesting that these microbial infections may play a critical role in the pathogenesis of many autoimmune diseases. Furthermore, it is interesting to note that the elimination of B cells bearing CD20 suppresses relapsing–remitting MS [39]. Interestingly, a study indicated that virus-infected B cells played a critical role in the pathogenesis of experimental autoimmune encephalomyelitis by inducing strong CNS antigen-reactive CD8^+^ T cells [88]. Therefore, further studies investigating the potential role of B cells in the pathogenesis of these autoimmune diseases, including animal model systems, would be crucial for future therapeutic applications with the cell population.

## Supplementary Materials

The following are available online at https://www.mdpi.com/2073-4409/9/8/1787/s1, Figure S1: Splenic B cells from SJL mice showed upregulated expression of CD80/86 costimulatory molecules and CD69 activation marker up on treatment with ligands of TLR2, TLR3, TLR4, TLR7, and TLR9.
