# Supplementary material for: Infection and Activation of B Cells by Theiler’s Murine Encephalomyelitis Virus (TMEV) Leads to Autoantibody Production in an Infectious Model of Multiple Sclerosis
Source: Cells. 2020 Jul 27;9(8):1787. doi: 10.3390/cells9081787 (PMC7465974; doi:10.3390/cells9081787)
Supplement: Supplementary file 1 [file cells-09-01787-s001.pdf]

Figure S1

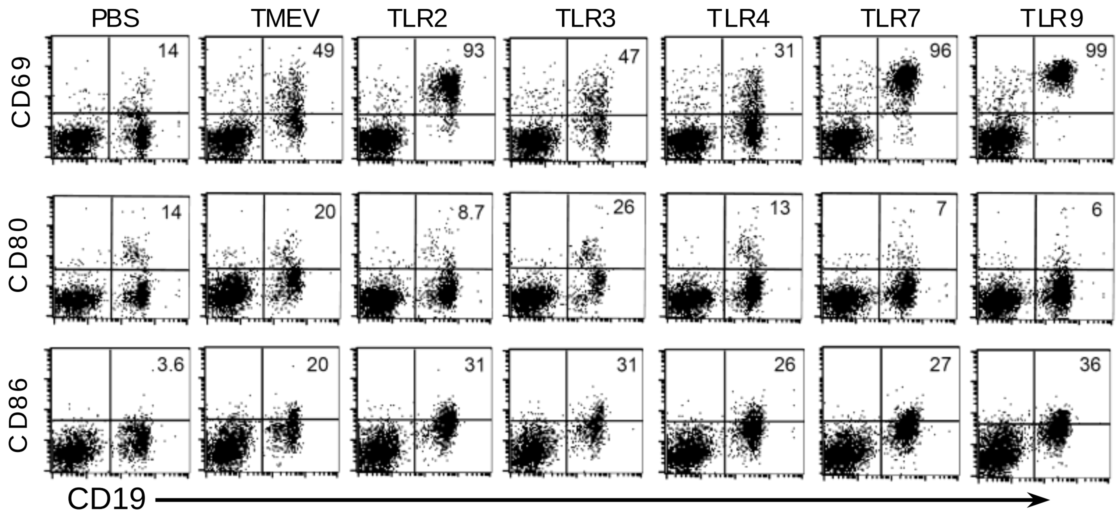

Figure S1. Splenic B cells from SJL mice showed upregulated expression of CD80/86 costimulatory molecules and CD69 activation marker up on treatment with ligands of TLR2, TLR3, TLR4, TLR7, and TLR9.
